# The mental health impact of the ongoing Russian-Ukrainian war 6 months after the Russian invasion of Ukraine

**DOI:** 10.3389/fpsyt.2023.1134780

**Published:** 2023-07-27

**Authors:** Anton Kurapov, Argyroula Kalaitzaki, Vladyslava Keller, Ivan Danyliuk, Tobias Kowatsch

**Affiliations:** ^1^Department of Experimental and Applied Psychology, Faculty of Psychology, Taras Shevchenko National University of Kyiv, Kyiv, Ukraine; ^2^Department of Social Work, Laboratory of Interdisciplinary Approaches to the Enhancement of Quality of Life, Health Sciences Faculty, Hellenic Mediterranean University, Crete, Greece; ^3^Faculty of Psychology, Taras Shevchenko National University of Kyiv, Kyiv, Ukraine; ^4^Institute for Implementation Science in Health Care, University of Zurich, Zurich, Switzerland; ^5^School of Medicine, University of St. Gallen, St. Gallen, Switzerland; ^6^Centre for Digital Health Interventions, Department of Management, Technology, and Economics at ETH Zurich, Zurich, Switzerland

**Keywords:** war, Ukraine, trauma experience, stress, anxiety, depression, PTSD, complex PTSD

## Abstract

**Objective:**

This study aimed to investigate the impact of the ongoing war in Ukraine on the mental health of Ukrainians, focusing on war-induced trauma, disturbances in self-organization, post-traumatic stress disorder, complex post-traumatic stress disorder, anxiety, stress, and depression.

**Methods:**

Data was collected from 703 participants 6 months after the full-scale invasion using a structured questionnaire that included sections on socio-demographic information, trauma-related issues, and mental health.

**Results:**

The study found that levels of depression and anxiety were relatively low, while stress and resilience were relatively high among Ukrainians affected by the war. However, those who were directly exposed to military actions, physical violence, or severe human suffering had higher levels of anxiety, depression, stress, and trauma-related symptoms. The war experience varied by gender, age, and living conditions. Participants who stayed in Ukraine had significantly lower anxiety, depression, stress, and trauma-related symptoms compared to those who moved abroad. Anxiety, depression, stress, low resilience, and subjective satisfaction with living conditions were predictors of trauma-related symptoms, including PTSD and CPTSD.

**Conclusion:**

These findings suggest that the mental health of Ukrainians affected by the war was impacted differently depending on their level of exposure to violence and their living conditions. Additionally, the study identified several predictors of trauma-related symptoms, including PTSD and CPTSD, such as anxiety, depression, stress, low resilience, and subjective satisfaction with living conditions. Future research should further explore the relationships between trauma type, sociodemographic factors, resilience, stress, anxiety, depression, and PTSD and CPTSD to better understand the mediation mechanisms underlying these relationships and to develop effective interventions to support the well-being of Ukrainians during this difficult time.

## 1. Introduction

On February 24th 2022, the Russian Federation started a full-scale invasion of Ukraine, causing a wide range of serious problems within the country and globally. A large body of research has shown that experiencing (witnessing) such traumatic events involving threat to one’s or other’s lives, or bodily integrity directly increases the risk of mental disorders (1–3): people with direct or indirect interaction with the war may develop serious physical and mental health issues. Within the context of war trauma, it is relevant to consider post-traumatic stress disorder (PTSD) and complex post-traumatic stress disorder (CPTSD) as indicators of trauma experience (4). The ICD-11 model of PTSD includes six symptoms measuring three core elements (each element is composed of two symptoms): (a) re-experiencing of the trauma in the present (Re), (b) avoidance of traumatic reminders (Av), and (c) a persistent sense of threat that is manifested by increased arousal and hypervigilance (Th). CPTSD is composed of six symptom clusters: three are shared with PTSD and three that are collectively referred to as “disturbances in self-organization” (DSO): affective dysregulation (AD), negative self-concept (NSC), and disturbed relationships (DR) (5).

The current study examines the impact that the war has had on the mental health of Ukrainians during the first 6 months, in particular, levels of anxiety, depression, stress, and trauma experience (prevailing trauma type, levels of PTSD, and CPTSD). This timeframe is of particular significance as it allows for an assessment of the immediate and ongoing effects of the conflict on mental health and by examining its multiple dimensions, the study provides a more nuanced and complete understanding of how the war is impacting Ukrainians. That is why we aim to investigate the following research hypotheses:

1. Exposure to the war in Ukraine is positively associated with higher levels of anxiety, depression, stress, DSP, PTSD, and CPTSD
2. Levels of anxiety, depression, stress, DSO, PTSD, and CPTSD differ based on gender, age, working conditions, and current exposure to traumatic experiences (placement within or outside Ukraine)
3. Anxiety, depression, stress, and resilience predict the severity of DSO, PTSD, and CPTSD.

## 2. Literature review

### 2.1. General impact of war on mental health

War and crisis are significant public health concerns with immense mental health implications. War-affected civilians are at a higher risk of developing mental health issues than military combatants (6). Anxiety, depression, and PTSD are the most prevalent mental health challenges reported among populations affected by war (7). About 86% of Syrians believe that war is the leading cause of their mental problems, having experienced unending war (8). Severe post-traumatic symptoms were observed among adult Palestinians who participated in the Great March of Return in the Gaza Strip (9). Moreover, exposure to war in Afghanistan has been associated with an increased prevalence of PTSD and suicidal attempts (10). Subsequently, people with a recent history of exposure to traumatic events are at a high risk of developing PTSD (8, 11). The impact of war on mental health could be long-lasting with lifelong implications (12, 13): survivors of World War II reportedly had a high prevalence of PTSD even more than 50 years after the war (13). Additionally, people in war-prone areas like Israel and Palestine are highly likely to suffer from prolonged adverse mental health effects like distress, anxiety, and depression (14).

### 2.2. Traumatic experience during the war

Wartime increases the exposure to traumatic events for the civilian population. Traumatic experiences during times of war and crisis mainly result in avoidance and re-experiencing of the events (8). Memories of traumatic events may trigger the development of PTSD. During war and crises, many people end up as internally displaced people (IDP) or refugees in neighboring nations. Unlike in their homes, the living conditions in refugee and IDP camps increase their predisposition to developing anxiety and depressive disorders (15). Conflict-induced displacements may also become significant traumatic experiences (15, 16). Forced displacements are characterized by heightened uncertainties among victims of war and crisis (17); uncertainties from forced evictions and loss of social support due to war increase the risk of developing anxiety and depression (18).

### 2.3. Stress and resilience

Stress is a normal body response to external perceived threats. Elevated stress levels are among the psychological impacts of the ongoing Russian invasion of Ukraine (19). Stress responses to traumatic events can be acute or chronic. Acute stress is characterized by intense emotional reactions to a traumatic event, usually within 1 month after exposure to the experience (20). However, some trauma survivors remain at risk of experiencing prolonged distress. Prolonged exposure to stressful events leads to severe mental health conditions like PTSD (21); stress due to exposure to violence (22) and loss of close family and friends (23) have adverse mental effects on war victims and civilians. High distress levels are reported among direct victims of war and those who have witnessed violence (22). Stress resilience has been observed in people from most countries confronted with war traumas. Shifting people’s focus from the war has helped to reduce the psychological burden associated with it: among Ukrainian refugees, attempts to stay and spend time with loved ones are the most common coping strategy against war-induced stress (24).

### 2.4. Anxiety, depression, and PTSD

Anxiety and depression are common responses by people from countries experiencing crisis and war (25–28). Anxiety levels may be high if people believe that the crisis will continue for a long time (28); high levels of depression and anxiety have been reported among young people exposed to war in Ukraine (23); elevated depression and increased tendencies toward alcohol and drug abuse have been reported among university students and personnel from Ukraine (29); a high prevalence of anxiety and depression has been reported among war refugees (15, 30, 31) and IDP (32). PTSD is common among people following traumatic experiences like war. Countries affected by war report high incidences of PTSD (11, 27, 33, 34): IDP and refugees are the primary victims of PTSD as they actively re-experience traumatic events (11).

## 3. Materials and methods

### 3.1. Data collection

The data was collected in one phase. The collection process started on July 22, 2022, and lasted until October 21, 2022. For this study, we used an online questionnaire (with the use of Google Forms) through a snowball procedure: the link to the questionnaire was shared through Telegram Channels with 5,000+ subscribers and instructions asked to share the survey with relatives and friends. Considering the diversity of subscribers, we managed to obtain information from people residing in Ukraine, and refugees, and even reached out to a few participants from currently occupied territories. All questions were presented in the Ukrainian language. The questionnaire form was divided into three main sections: socio-demographic, trauma-related variables, and mental health.

### 3.2. Study design and measures

For this research, we have used a descriptive-correlational design. Dependent variables of this study included: depression, anxiety, stress, resilience, DSO, PTSD, and CPTSD; while independent variables included gender, age, satisfaction with current living conditions, and current exposure to traumatic experience (placement within or outside Ukraine). For defining predictors of PTSD, DSO, and CPTSD (dependent variables) we have used depression, anxiety, stress, and resilience (independent variables) in order to identify whether the latter could increase the severity of trauma experience symptoms.

*Sociodemographic data*: Information about the sociodemographic data included standardized questions that concerned several aspects (see Table 1).

**Table 1:** Overview of sociodemographic data.

		*N*	%
Gender	Male	155	22.0
	Female	548	78.0
Marital status	Single	275	39.1
	Married	364	51.8
	Divorced	56	8.0
	Widowed	8	1.1
Education	Doctorate	54	7.7
	Higher (Masters)	466	66.3
	Secondary	114	16.2
	Incomplete Higher	2	0.3
	Professional	46	6.5
	Incomplete Secondary	2	0.3
	Other	18	2.6
Children	No	442	62.9
	1	140	19.9
	2	107	15.2
	3	13	1.8
	5	1	0.1
Job	Unemployed	126	17.9
	Governmental worker	34	4.8
	Regular employee	218	31.0
	Freelancer/self-employed/entrepreneur	134	19.1
	Retired	12	1.7
	Student	171	24.3
	Other	8	1.1
Working conditions	Online	229	32.6
	Offline	179	25.5
	Unemployed	295	42.0
Financial state	Not enough for basic needs	50	7.1
	Enough for basic needs and additional needs	25	3.6
	Enough only for basic needs	208	29.6
	Enough money for all needs	132	18.8
	Enough money for basic and some additional needs	288	41.0

*Traumatic events, DSO, PTSD, and CPTSD*: The exposure to traumatic events was measured by the Life Events Checklist (LEC-5 (35)). To assess trauma exposure, we have used the International Trauma Questionnaire (ITQ-9 (36)) (Cronbach’s alpha = 0.83, 0.84, and 0.74 for PTSD, DSO, and CPTSD, respectively). We have differentiated the most prevailing trauma types into the following categories: (i) experience of war (war exposure), (ii) death of a close person/relative, (iii) accident, (iv) military actions, (v) enemy occupation, (vi) sexual violence, (vii) rocket attacks, (viii) family problems, (ix) personal problems, (x) physical injury, (xi) road accident.

*Mental health assessment*: For the assessment of resilience, The Brief Resilience Scale (BRS-6 (37)) with 6 items was used (Cronbach’s alpha = 0.81). The perceived stress level was measured with The Perceived Stress Scale-4 (PSS-4 (38)) (Cronbach’s alpha = 0.71). Depression and anxiety were measured with The Patient Health Questionnaire for Depression and Anxiety (PHQ-9 (39)) (Cronbach’s alpha is 0.84 for anxiety and 0.81 for depression, respectively).

### 3.3. Participants

The inclusion criteria for the participants were: age 18–65 and the ability to give informed consent. The exclusion criterion concerned the issue of accessibility: inability to access internet or being under censorship (i.e., on the occupied territories). The total number of participants that fulfilled the criteria was 703 (age *M* = 32.1, *SD* = 12.1), 77.9% female. A detailed overview of sociodemographic data is presented in Table 1.

We consider the current location of the participant to be important in the perception of trauma and the overall level of traumatization. The following major categories have been identified: no movement, movement inside and outside of the country. The first category included three major cases: (i) stayed in the same place that was never occupied, (ii) stayed in the same place and it was occupied, (iii) stayed in the same place and it was de-occupied. The second category included two major cases: (i) moved to another place in Ukraine and did not register as an official IDP, (ii) moved to another place in Ukraine and officially registered as IDP. The third category included two cases: (i) moved to another country and did not officially register as a refugee, (ii) moved to another country and registered as a refugee. Detailed differentiation is presented in Table 2.

**Table 2:** Differentiation of current location.

Current location	*N*	%
**Within Ukraine**		
The same place as before the war, never occupied	380	54.1%
Moved within Ukraine, officially registered IDP	56	8.0%
Moved within Ukraine, did not officially register as IDP	52	7.4%
The same place under occupation now	7	1.0%
**Outside Ukraine**
Moved to another country	163	23.2%
Moved to another country, did not officially register as a refugee	8	1.1%

### 3.4. Statistical analysis

Statistical analysis was conducted using R (version 2022.07.1) and Jamovi (version 2.3.18). (i) To identify an overall level of traumatization and the prevailing type of trauma, we have used descriptive statistics, such as mean, standard deviations, frequencies, t-test, and one-way ANOVA. According to the power estimates, to detect a moderate effect size of 0.5 with a desired power of 0.80 and an alpha level of 0.05, for one-way ANOVA we need a sample size of 63 respondents per each subgroup of a fixed factor (*N* = [2*(*Z*_α/2 + *Z*_*β*)^2*σ^2]/Δ^2, where *N* is the required sample size, *Z*_α/2, and *Z*_*β* are the critical values of the standard normal distribution for the desired significance level (α) and power (1-*β*), respectively, σ^2 is the population variance, and Δ is the effect size). (ii) To identify the statistically significant mean differences with gender, age, working conditions, and current exposure to traumatic experience (placement within or outside Ukraine) as fixed factors we have used one-way ANOVA with the same power estimates. (iii) To identify whether anxiety, depression, stress, and resilience are the predictors of DSO, PTSD, and CPTSD, we have used correlation analysis (Pearson), linear regression, and generalized linear modeling together with the estimation of the effect size (epsilon squared). According to the power estimates, to detect a large effect size of *R*^2^ = 0.6 with a desired power of 0.80 and an alpha level of 0.05 for multiple linear regression with two predictor variables, we need a sample of at least 71 participants (*N* = [*F*(*k*, *N* – *k* − 1)*(1 − *R*^2)/*R*^2] + *k*, where *k* is the number of predictor variables, *N* is the total sample size, *R*^2^ is the proportion of variance in the outcome variable explained by the predictor variables, and *F*(*k*, *N* – *k* − 1) is the critical *F*-value for a given significance level and degrees of freedom). Based on power estimates, obtained sample size of 703 participants is representative of the populations from which the study subjects were drawn and is sufficient obtain desired statistical effects.

## 4. Results

### 4.1. Overall level of traumatization

#### 4.1.1. Mean scores

Results show that Ukrainians, after 6 months of the war, show low levels of depression (*M* = 2.45, *SD* = 1.80; score range 0–5), and low levels of anxiety (*M* = 2.26, *SD* = 1.80; score range 0–5). At the same time, levels of stress (*M* = 7.55, *SD* = 3.16; score range 0–16) and resilience (*M* = 2.85, *SD* = 0.84; score range 1–5) are relatively high. The levels of traumatization are the following: PTSD (*M* = 10.95, *SD* = 6.20; score range 0–30), DSO (*M* = 11.59, *SD* = 7.65; score range 0–30), and CPTSD (*M* = 22.6, *SD* = 11.9; score range 0–60).

#### 4.1.2. Traumatic life events

**Figure 1:**
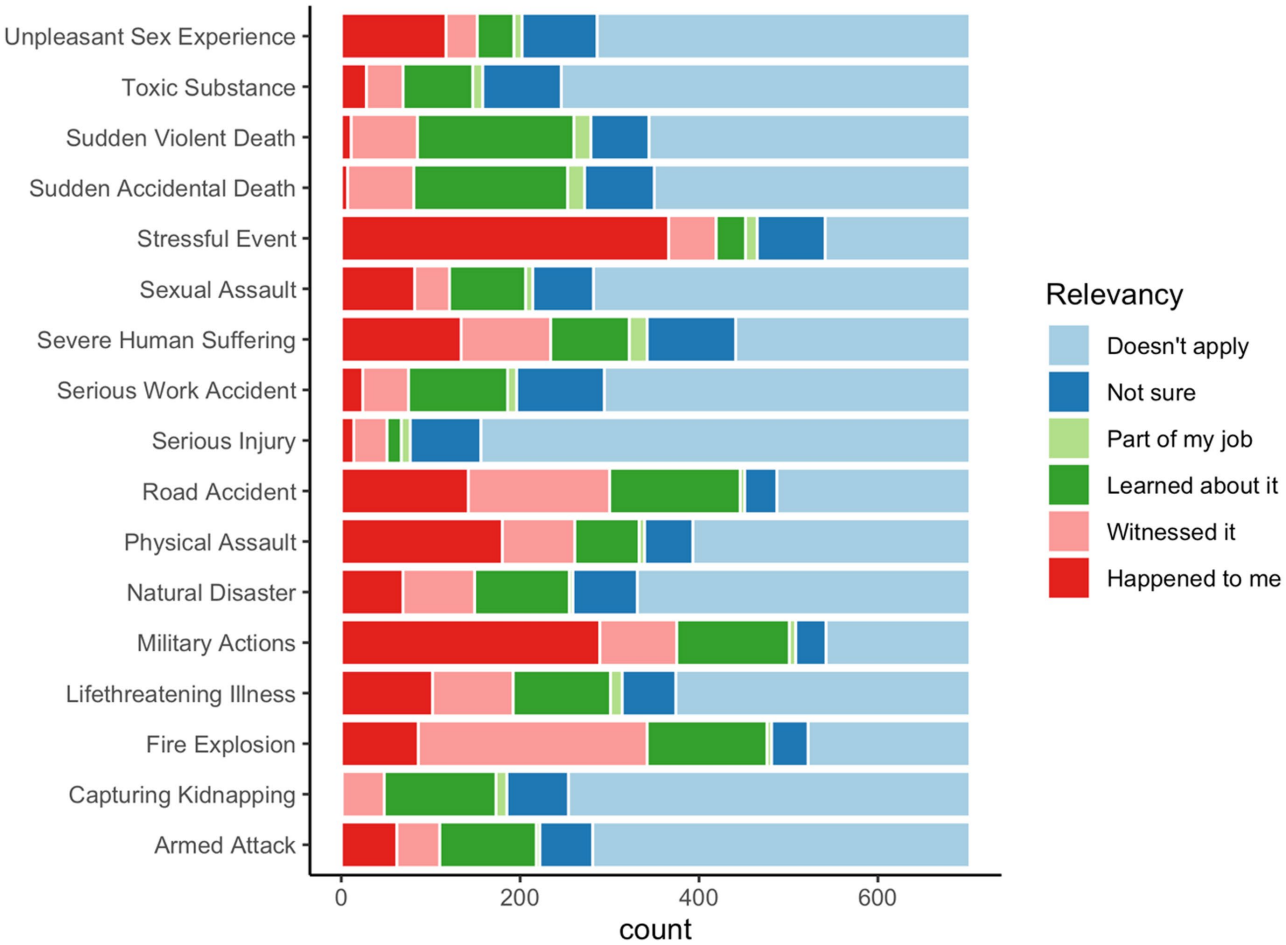
Frequency and relevancy of traumatic life events according to LEC-5.

Traumatic events that happened with participants were reported through LEC-5 (see Figure 1) and ITQ-9 (see Table 3). Based on LEC-5 data, the most prevailing traumatic life events that happened with participants include: other stressful events (*N* = 366), military actions (*N* = 289), physical assault (*N* = 180), road accidents (*N* = 142), and severe human suffering (*N* = 134). The frequency distribution of traumatic events is presented in Figure 1.

**Table 3:** Mean scores of depression, and DSO according to the type of trauma.

**Trauma type**	***N***	**%**	**Depression**		**DSO**		**CPTSD**	
			**Mean**	**SD**	**Mean**	**SD**	**Mean**	**SD**
War	316	46.6%	2.41	1.81	11.53	7.58	22.1	11.57
Death of a relative	96	14.2%	2.63	1.85	11.73	7.5	23.4	11.83
Accident	49	7.2%	2.65	1.82	11.41	7.64	22.6	10.52
Military actions	36	5.3%	2.22	1.60	13.78	8.22	25.6	25.6
Occupation	29	4.3%	2.45	1.76	12.21	6.24	23.0	12.75
Sexual violence	23	3.4%	2.35	1.71	10.13	6.53	23.3	9.11
Rocket attacks	19	2.8%	2.26	1.75	9.89	7.64	21.3	11.82
Family problems	19	2.8%	2.68	2.03	11.58	6.64	21.6	9.54
Personal problems	17	2.5%	2.47	1.08	13.47	9.55	24.8	14.85
Physical injury	15	2.2%	2.27	1.90	8.93	7.39	20.8	13.03
Road accident	11	1.6%	2.82	1.99	12.36	7.54	21.9	11.21
Absent	48	7.1%	2.35	1.88	10.31	8.01	20.3	14.08

The results of one-way ANOVA show that participants who were directly exposed to military actions, witnessed them, or learned about them show higher levels of anxiety (*F* (5, 56.5) = 2.78, *p* = 0.026, ω^2^ = 0.04), PTSD (*F* (5, 56.7) = 4.06, *p* = 0.003, ω^2^ = 0.03), and CPTSD (*F* (5, 55.4) = 2.09, *p* = 0.080, ω^2^ = 0.01). The same concerns participants, who reported experiencing “other stressful event,” which, as we assume, is war-related: they show higher levels of anxiety (*F* (5, 83.5) = 10.93, *p* < 0.001, ω^2^ = 0.06), depression (*F* (5, 84.3) = 10.46, *p* < 0.001, ω^2^ = 0.06), stress (*F* (5, 83.0) = 5.09, *p* < 0.001, ω^2^ = 0.03), DSO (*F* (5, 84.2) = 14.6, *p* < 0.001, ω^2^ = 0.09), PTSD (F (5, 84.2) = 16.66, *p* < 0.001, ω^2^ = 0.11), and CPTSD (*F* (5, 84.4) = 20.29, *p* < 0.001, ω^2^ = 0.14). Those who reported exposure to physical violence show higher levels of anxiety (*F* (5, 50) = 4.33, *p* = 0.002, ω^2^ = 0.02), depression (*F* (5, 51.7) = 8.52, *p* < 0.001, ω^2^ = 0.02), DSO (*F* (5, 49.9) = 3.62, *p* = 0.007, ω^2^ = 0.01), PTSD (*F* (5, 53.1) = 3.75, *p* < 0.006, ω^2^ = 0.01), and CPTSD (*F* (5, 50.9) = 5.13, *p* < 0.001, ω^2^ = 0.02). Participants reporting the experience of severe human suffering show the highest differences in mean scores in anxiety (*F* (5, 144) = 10.68, *p* < 0.001, ω^2^ = 0.06), depression (*F* (5, 145) = 10.13, *p* < 0.001, ω^2^ = 0.06), stress (*F* (5, 147) = 5.98, *p* < 0.001, ω^2^ = 0.04), DSO (*F* (5, 149) = 8.24, *p* < 0.001, ω^2^ = 0.06), PTSD (*F* (5, 150) = 7.18, *p* < 0.001, ω^2^ = 0.05), and CPTSD (F (5, 150) = 10.43, *p* < 0.001, ω^2^ = 0.07). The differences in mean scores are presented in Tables A1–A4 in the Appendix A.

#### 4.1.3. Prevailing trauma type

According to reports from ITQ-9, the beginning of the war, war exposure, or the war itself became the most traumatic event for the majority of Ukrainians (46.6%). No statistically significant differences among different trauma types (as fixed factor) and anxiety (*F* (16, 35.1) = 0.92, *p* = 0.551), stress (*F* (16, 34.8) = 0.95, *p* = 0.521), PTSD (*F* (16, 34.9) = 0.58, *p* = 0.876), or resilience (*F* (16, 35.2) = 0.51, *p* = 0.925) were obtained; however, significant differences were obtained for depression (*F* (16, 35.4) = 2.191, *p* = 0.026), DSO (*F* (16, 36.9) = 10.365, *p* < 0.001), and CPTSD (*F* (16, 38.9) = 9.3, *p* < 0.001). Mean scores of depression, stress, DSO, and CPTSD by the trauma type are presented in Table 3.

### 4.2. Sociodemographic factors

#### 4.2.1. Gender and age

Compared to men, women had higher levels of anxiety (*M* = 1.69, *SD* = 1.64 vs. *M* = 2.42, *SD* = 1.69; *t* = −4.70, *p* < 0.01, Cohen’s *d* = −0.44), depression (*M* = 2.59, *SD* = 1.81 vs. *M* = 1.98, *SD* = 1.68; *t* = −3.66, *p* < 0.01, Cohen’s *d* = −0.34), and stress (*M* = 7.77, *SD* = 3.1 vs. *M* = 6.7, *SD* = 3.33; *t* = −3.67, *p* < 0.01, Cohen’s *d* = −0.34); while men showed higher levels of resilience (*M* = 3.31, *SD* = 0.83) than women (*M* = 2.73, *SD* = 0.8; *t* = 7.67, *p* < 0.01, Cohen’s *d* = 0.71). Women also show higher levels of DSO (*t* = −2.57, *p* = 0.01; *M* = 12.00, *SD* = 7.76, Cohen’s *d* = −0.24) than men (*M* = 10.23, *SD* = 7.09), PTSD (*t* = −3.09, *p* = 0.002, Cohen’s *d* = −0.29; *M* = 11.40, *SD* = 6.16 for women and *M* = 10.23, *SD* = 7.14 for men) and CPTSD (*t* = −3.27, *p* = 0.001, Cohen’s *d* = −0.31; *M* = 23.45, *SD* = 11.87 for women and *M* = 19.87, *SD* = 11.62 for men).

Adults at the age between 28 and 45 show the highest levels of anxiety (*M* = 2.49, *SD* = 1.67; *F* (3) = 5.20, *p* < 0.001, ω^2^ = 0.02), depression (*M* = 2.75, *SD* = 1.64; *F* (3) = 3.53, *p* = 0.015, ω^2^ = 0.01), DSO (*M* = 13.48, *SD* = 7.37; *F* (3) = 8.78, *p* < 0.001, ω^2^ = 0.03), PTSD (*M* = 12.07, *SD* = 5.96; *F* (3) = 3.13, *p* < 0.001, ω^2^ = 0.03), and CPTSD (*M* = 25.56, *SD* = 11.11; *F* (3) = 8.06, *p* < 0.001, ω^2^ = 0.04), while participants at the age of 18–27 (*M* = 3.07, *SD* = 0.88) and 46–60 (*M* = 2.96, *SD* = 0.81) have highest levels of resilience (*F* (3) = 4.80, *p* = 0.003, ω^2^ = 0.02). Interaction between age and gender as fixed factors does not show statistical significance in the obtained models.

#### 4.2.2. Job, living conditions, and current location

Employment is related to satisfaction with current living conditions (*χ*^2^ = 38.7, *df* = 8, *p* < 0.029), while the latter is capable of defining the trauma experience. As such, the better the reported living conditions, the lower are the levels of anxiety (*F* (4, 79.3) = 5.58, *p* < 0.001), depression (*F* (4, 80.9) = 17.27, *p* < 0.001), stress (*F* (4, 80.2) = 18.32, *p* < 0.001), DSO [*F* (4, 79.6) = 15.61, *p* < 0.001], PTSD (*F* (4, 78) = 4.64, *p* = 0.002), and CPTSD (*F* (4, 79) = 13.57, *p* < 0.001). The difference in mean scores is presented in Table A5 in the Appendix. Ukrainians who were forced to move either abroad or become IDP show higher levels of stress (*F* (7, 49.6) = 2.6, *p* < 0.002), DSO (*F* (7, 49.4) = 2.62, *p* < 0.022), and PTSD (*F* (7, 49.3) = 2.22, *p* < 0.048). Proximity to the war zone does not have an effect on the levels of anxiety, depression, DSO, PTSD, and CPTSD. The difference in mean scores is presented in Table A6 in the Appendix.

We also have divided the sample into two major groups: those who stayed in Ukraine, and those who moved abroad. A t-test was conducted to compare the mean scores on anxiety between those who stayed (*M* = 2.18, *SD* = 1.67) and those who moved abroad (*M* = 2.49, *SD* = 1.78). The results showed a statistically significant difference between the two groups (*t* = −2.07, *df* = 701, *p* < 0.05), with those who stayed showing significantly lower levels of anxiety than those who moved abroad. Identical results have been obtained for: depression (*t* = −2.22, *df* = 701, *p* < 0.05) with those who stayed showing significantly lower levels of depression (*M* = 2.36, *SD* = 1.17) than those who moved abroad (*M* = 2.71, *SD* = 1.92); stress (*t* = −2.99, *df* = 701, *p* < 0.05) with those who stayed showing significantly lower levels of stress (*M* = 7.35, *SD* = 3.19) than those who moved abroad (*M* = 8.16, *SD* = 2.95); DSO (*t* = −2.82, *df* = 701, *p* < 0.05) with those who stayed showing significantly lower levels of DSO (*M* = 11.13, *SD* = 7.46) than those who moved abroad (*M* = 13.01, *SD* = 8.07); and resilience (*t* = 6.31, *df* = 701, *p* < 0.05) with those who stayed showing significantly higher levels of resilience (*M* = 2.96, *SD* = 0.86) than those who moved abroad (*M* = 2.51, *SD* = 0.66).

#### 4.2.3. Sociodemographic factors and trauma exposure

No statistically significant differences were obtained while performing two-way ANOVA for investigating effects of gender, reported traumatic events according to LEC-5 (any stressful event, military actions, physical violence, severe human suffering), the interaction of both fixed factors on anxiety, depression, stress, DSO, PTSD, and CPTSD. Age as a fixed factor in interaction with reported traumatic events according to LEC-5 also did not allow to obtain statistically significant results. While performing two-way ANOVA, gender, age, and decision to leave or stay in Ukraine did not show statistically significant interaction with reported traumatic events according to LEC-5 and on anxiety, depression, stress, DSO, PTSD, and CPTSD as dependent variables.

### 4.3. Predictors of trauma experience

#### 4.3.1. Anxiety and depression

Anxiety positively correlates with DSO (*r* = 0.462, *p* < 0.001), PTSD (*r* = 0.594, *p* < 0.001), and CPTSD (*r* = 0.510, *p* < 0.001); depression positively correlates with DSO (*r* = 0.576, *p* < 0.001), PTSD (*r* = 0.359, *p* < 0.001), and CPTSD (*r* = 0.558, *p* < 0.001). Increased anxiety and depression levels are the predictors of DSO (*R*^2^ = 0.351; Intercept: *B* = 4.985, *SE* = 0.427, *t* = 11.68, *p* < 0.001) and together with the subjective satisfaction with living conditions are the predictors of CPTSD (*R*^2^ = 0.379; Intercept: *B* = 10.47, *SE* = 1.117, *t* = 9.37, *p* < 0.001).

#### 4.3.2. Resilience and stress

Whereas stress is positively correlated with DSO (*r* = 0.594, *p* < 0.001), PTSD (*r* = 0.376, *p* < 0.001), and CPTSD (*r* = 0.579, *p* < 0.001), resilience is negatively correlated with DSO (*r* = −0.528, *p* < 0.001), PTSD (*r* = −0.303, *p* < 0.001), and CPTSD (*r* = −0.498, *p* < 0.001). Increased stress and reduced resilience levels, together with subjective satisfaction with living conditions, are the predictors of PTSD (*R*^2^ = 0.408; Intercept: *B* = 11.331, *SE* = 1.986, *t* = 5.704, *p* < 0.001), DSO (*R*^2^ = 0.447; Intercept: *B* = 14.578, *SE* = 1.99, *t* = 7.304, *p* < 0.001), and CPTSD (*R*^2^ = 0.642; Intercept: *B* = 25.909, *SE* = 3.194, *t* = 8.111, *p* < 0.001).

## 5. Discussion

### 5.1. Exposure to the war trauma

Despite a number of studies showing that war has a devastating impact on the mental health of the population exposed to war (7, 8, 10, 40, 41), our study showed that Ukrainians, after 6 months of experiencing war, show relatively low scores of anxiety and depression, and medium levels of DSO, PTSD, and CPTSD. High scores have been obtained on acute stress and resilience levels, which explain the low levels of DSO, PTSD, and CPTSD (42). However, the same results do not concern the participants who have been directly exposed to war trauma (those who reported experiencing military attacks, physical violence, and severe human suffering), the number of which does not exceed 25% from the entire sample, meaning that despite reporting the war itself as the most traumatic experience and witnessing it, Ukrainians still show relatively low levels of anxiety, depression, stress, DSO, PTSD, and CPTSD in comparison to the sample average. Overall, participants who have been witnessing military actions or experiencing physical violence, together with severe human suffering, just have higher levels of anxiety, depression, stress, DSO, PTSD, and CPTSD than those who did not report having such experience, but at the same time, not critically high. Higher levels of depression are more common for respondents who experienced personal trauma (e.g., non war-related deaths of parents or relatives), while higher levels of DSO, PTSD, and CPTSD are more common for war-related trauma experiences.

We assume that the low levels of anxiety and depression among Ukrainians can be explained by a large amount of perceived social support. Previous studies have shown that in the conditions of war, Ukrainian society feels more united than ever (43). The current situation is unique compared to earlier studies of local conflicts, which typically involve isolated combatants and refugees (e.g., on a state border) (44). Ukrainians can share their experiences and receive support and understanding at all levels, from households to the state. This support may lead to high levels of resilience, which are common for all Ukrainians.

Since most of the studies concerning the traumatic impact of war have been conducted mainly during the post-war period, we assume that the situation may change as soon as the acute phase of the war will be over. According to a longitudinal study on Israeli civilians’ mental health during the Israel-Gaza 2008–2009 war (45), perceived social support moderated the decrease in PTSD, anxiety, and depression symptoms over time. Nevertheless, Ukrainians reported that the war itself became a major traumatic event for the majority of the population. In addition, we have identified the prevailing trauma types: (i) experience of war (war exposure), (ii) death of a close person/relative, (iii) accident, and (iv) military actions, which comply with the reported trauma events that include other stressful event (we assume this is war-related), military actions, physical assault, road accident, and severe human suffering. The model of polyvictimization (46) suggests that experience of multiple types of victimization over time leads to increased mental health problems and since Ukrainians, based on the results of our study and on the open facts, unergo polyvictimization, overall situation with mental health may worsen. However, it is also possible that the low levels of stress, depression, and PTSD among Ukrainians could be due to a lack of awareness acknowledgment of these issues. Many people may not realize that they are experiencing stress or mental health problems, or they may not seek out help for these issues due to stigma or other barriers. It is possible that the prevalence of stress, depression, and PTSD in Ukraine is actually higher than reported, but that these issues are not being adequately addressed.

### 5.2. Demographics

Women are more prone to traumatic experiences and increased levels of anxiety, depression, DSO, PTSD, and CPTSD, which was proven by other studies as well (47). Younger (up to 25) and older (over 46) participants show lower levels of trauma experience, while adults (aged between 26 and 45) show higher levels of trauma experience. Because of the war, many Ukrainians lost their jobs, and it impacted their financial state, either while staying in Ukraine or while fleeing to other countries. Half of the respondents maintain their job duties either online (remotely) or offline. The current financial state does not depend on whether people flee from or stay in Ukraine. The financial state majorly defines the current, subjectively reported living comfort, which relates to the trauma experience: the worse the subjective living conditions are, the higher the levels of stress, depression, DSO, PTSD, and CPTSD.

### 5.3. Stress, resilience, anxiety, and depression

Acute stress increases the risk of developing PTSD, while increased resilience reduces the level of trauma experience. It has been proven by several studies (20, 25, 48, 49). Results have shown that Ukrainians are ready to adapt to new conditions (living in the state of war) and are open to acceptance and development of new lifestyles, show increased confidence, self-reliance and develop meaningful narratives to live through the hard times. Despite that, regression models showed that due to the presence of increased stress, anxiety, and depression, together with reduced resilience, the current situation may change for the worse in the future, especially if current living conditions are not perceived as satisfactory. In particular, increased levels of DSP, PTSD, and CPTSD symptoms are to be expected. It means that when the war ends, all Ukrainians will develop certain difficulties in self-perception and in relations with other people due to exposure to acute stress and war-related trauma. What is more, the stress-diathesis model (50) suggests that mental health outcomes are the result of both environmental stressors and individual vulnerabilities, meaning that being exposed to war-related trauma, together with increased individual anxiety and stress, will lead to worse mental health outcomes after the direct exposure is over, in particular, DSO and prolonged depression.

Anxiety and depression play are critical for traumatic experiences. As such, anxiety and depression correlate with DSO, PTSD, and CPTSD, meaning that more anxious and depressed individuals will experience more severe trauma-related consequences. For example, this has already been shown in studies with Cambodian refugees (51). Multiple traumatizations, which apply to the Ukrainian sample, might further affect the emergence of clinical depression (52). In addition, increased depression makes a person more exposed to traumatic experiences (53). Nevertheless, despite being traumatized, Ukrainians did not yet show severe signs of serious consequences of traumatization.

## 6. Limitations

One of the greatest limitations of this study is its inability to reach individuals of the Ukrainian population who experienced a direct impact of war, for example, those who live on occupied territories, those who have been forced to move to the Russian Federation, or those who have experienced direct physical or sexual violence. This study reports on the general population of Ukraine that mainly lives on the territories that have never been occupied or who have escaped from war during the first several months after its beginning. To this end, further investigations are planned to understand better individuals in the territories that have been de-occupied and/or those who have returned after forced displacement since we presume that those populations would have more severe levels of traumatization. And, of course, it is necessary to distinguish between civilian and militant Ukrainians. The research sample has a relatively low number of men, in comparison to women, which may be caused by the inability to reach out to male population due to direct engagement in military activities (or service in the army) and this is another limitation of the current study. Other limitations are consistent with the disadvantages of using the snowball method for sampling. This may include the inability to control the sampling process, the weight of the first respondents which leads to community bias, and also lack of representativeness due to the non-random way of population sampling. Besides, the cross-sectional study design implies that data are collected at a single moment in time, whereas data collection for this study lasted for almost 4 months.

Despite all these limitations, the data of this work is an important building block to inform the design of scalable (digital) public health interventions for individuals living in crises that explicitly target mental health (54, 55).

## Data availability statement

The raw data supporting the conclusions of this article will be made available by the authors, without undue reservation.

## Ethics statement

The studies involving human participants were reviewed and approved by Ethics Committee of the Hellenic Mediterranean University (87/17-10-2022). The patients/participants provided their written informed consent to participate in this study.

## Author contributions

ArK: conceptualization and methodology of the study. AnK and ID: data collection and data proofing. AnK and VK: data analysis. AnK, VK, and TK: manuscript writing. ArK, AnK, and TK: manuscript review. All authors contributed to the article and approved the submitted version.

## Conflict of interest

TK is affiliated with the Centre for Digital Health Interventions (CDHI), a joint initiative of the Institute for Implementation Science in Health Care, University of Zurich, the Department of Management, Technology, and Economics at ETH Zurich, and the Institute of Technology Management and School of Medicine at the University of St. Gallen. CDHI is funded in part by CSS, a Swiss health insurer. TK is also a co-founder of Pathmate Technologies, a university spin-off company that creates and delivers digital clinical pathways. However, neither CSS nor Pathmate Technologies was involved in this research.

The remaining authors declare that the research was conducted in the absence of any commercial or financial relationships that could be construed as a potential conflict of interest.

## Publisher’s note

All claims expressed in this article are solely those of the authors and do not necessarily represent those of their affiliated organizations, or those of the publisher, the editors and the reviewers. Any product that may be evaluated in this article, or claim that may be made by its manufacturer, is not guaranteed or endorsed by the publisher.
