# Supplementary material for: The mental health impact of the ongoing Russian-Ukrainian war 6 months after the Russian invasion of Ukraine
Source: Front Psychiatry. 2023 Jul 27;14:1134780. doi: 10.3389/fpsyt.2023.1134780 (PMC10412819; doi:10.3389/fpsyt.2023.1134780)
Supplement: Supplementary file 1 [file Table_1.docx]

**Appendix**

Table A1

Mean Differences for Anxiety, PTSD, and CPTSD according to the Witnessing Military Actions

|  | | **LEC 5 military_actions** | | **N** | | **Mean** | | **SD** | | **SE** | |
| --- | --- | --- | --- | --- | --- | --- | --- | --- | --- | --- | --- |
| Anxiety |  | not about me |  | 157 |  | 2.140 |  | 1.80 |  | 0.144 |  |
|  |  | not sure |  | 34 |  | 2.471 |  | 2.08 |  | 0.356 |  |
|  |  | got to know |  | 123 |  | 2.228 |  | 1.56 |  | 0.141 |  |
|  |  | work |  | 7 |  | 0.857 |  | 1.07 |  | 0.404 |  |
|  |  | witnessing |  | 83 |  | 2.205 |  | 1.67 |  | 0.183 |  |
|  |  | happened with me |  | 277 |  | 2.379 |  | 1.68 |  | 0.101 |  |
| PTSD |  | not about me |  | 157 |  | 9.204 |  | 6.46 |  | 0.515 |  |
|  |  | not sure |  | 34 |  | 11.647 |  | 5.90 |  | 1.012 |  |
|  |  | got to know |  | 123 |  | 11.000 |  | 5.41 |  | 0.488 |  |
|  |  | work |  | 7 |  | 8.571 |  | 3.78 |  | 1.429 |  |
|  |  | witnessing |  | 83 |  | 11.723 |  | 7.01 |  | 0.770 |  |
|  |  | happened with me |  | 277 |  | 11.809 |  | 6.05 |  | 0.364 |  |
| CPTSD |  | not about me |  | 157 |  | 19.904 |  | 12.20 |  | 0.974 |  |
|  |  | not sure |  | 34 |  | 23.706 |  | 12.13 |  | 2.080 |  |
|  |  | got to know |  | 123 |  | 23.350 |  | 10.51 |  | 0.947 |  |
|  |  | work |  | 7 |  | 21.286 |  | 11.10 |  | 4.196 |  |
|  |  | witnessing |  | 83 |  | 24.060 |  | 13.20 |  | 1.449 |  |
|  |  | happened with me |  | 277 |  | 23.415 |  | 11.75 |  | 0.706 |  |
|  | | | | | | | | | | | |

Table A2

Mean Differences for Anxiety, Depression, Resilience, Stress, DSO, PTSD, and CPTSD according to the Reporting of Experiencing Stressful Event

|  | | **LEC 5 any stressful event** | | **N** | | **Mean** | | **SD** | | **SE** | |
| --- | --- | --- | --- | --- | --- | --- | --- | --- | --- | --- | --- |
| Anxiety |  | not about me |  | 155 |  | 1.71 |  | 1.546 |  | 0.1242 |  |
|  |  | not sure |  | 71 |  | 1.59 |  | 1.316 |  | 0.1561 |  |
|  |  | got to know |  | 33 |  | 2.58 |  | 1.562 |  | 0.2719 |  |
|  |  | work |  | 13 |  | 1.77 |  | 1.739 |  | 0.4824 |  |
|  |  | witnessing |  | 51 |  | 2.10 |  | 1.404 |  | 0.1965 |  |
|  |  | happened with me |  | 358 |  | 2.65 |  | 1.788 |  | 0.0945 |  |
| Depression |  | not about me |  | 155 |  | 1.90 |  | 1.835 |  | 0.1474 |  |
|  |  | not sure |  | 71 |  | 1.90 |  | 1.465 |  | 0.1739 |  |
|  |  | got to know |  | 33 |  | 2.85 |  | 1.523 |  | 0.2652 |  |
|  |  | work |  | 13 |  | 1.85 |  | 1.463 |  | 0.4058 |  |
|  |  | witnessing |  | 51 |  | 1.90 |  | 1.540 |  | 0.2156 |  |
|  |  | happened with me |  | 358 |  | 2.87 |  | 1.806 |  | 0.0955 |  |
| Resilience |  | not about me |  | 155 |  | 3.12 |  | 0.859 |  | 0.0690 |  |
|  |  | not sure |  | 71 |  | 2.96 |  | 0.827 |  | 0.0981 |  |
|  |  | got to know |  | 33 |  | 3.03 |  | 0.729 |  | 0.1269 |  |
|  |  | work |  | 13 |  | 3.05 |  | 0.740 |  | 0.2053 |  |
|  |  | witnessing |  | 51 |  | 3.04 |  | 0.794 |  | 0.1112 |  |
|  |  | happened with me |  | 358 |  | 2.68 |  | 0.823 |  | 0.0435 |  |
| Stress |  | not about me |  | 155 |  | 6.55 |  | 3.483 |  | 0.2798 |  |
|  |  | not sure |  | 71 |  | 7.49 |  | 2.990 |  | 0.3548 |  |
|  |  | got to know |  | 33 |  | 7.24 |  | 2.359 |  | 0.4106 |  |
|  |  | work |  | 13 |  | 7.23 |  | 3.492 |  | 0.9684 |  |
|  |  | witnessing |  | 51 |  | 7.02 |  | 2.970 |  | 0.4159 |  |
|  |  | happened with me |  | 358 |  | 8.08 |  | 3.017 |  | 0.1595 |  |
| DSO |  | not about me |  | 155 |  | 7.76 |  | 7.165 |  | 0.5755 |  |
|  |  | not sure |  | 71 |  | 11.65 |  | 7.184 |  | 0.8525 |  |
|  |  | got to know |  | 33 |  | 12.00 |  | 6.320 |  | 1.1001 |  |
|  |  | work |  | 13 |  | 11.08 |  | 6.739 |  | 1.8690 |  |
|  |  | witnessing |  | 51 |  | 9.55 |  | 6.718 |  | 0.9407 |  |
|  |  | happened with me |  | 358 |  | 13.63 |  | 7.546 |  | 0.3988 |  |
| PTSD |  | not about me |  | 155 |  | 7.79 |  | 6.075 |  | 0.4880 |  |
|  |  | not sure |  | 71 |  | 9.68 |  | 4.828 |  | 0.5730 |  |
|  |  | got to know |  | 33 |  | 10.88 |  | 5.355 |  | 0.9321 |  |
|  |  | work |  | 13 |  | 8.69 |  | 4.347 |  | 1.2057 |  |
|  |  | witnessing |  | 51 |  | 10.67 |  | 6.689 |  | 0.9367 |  |
|  |  | happened with me |  | 358 |  | 12.82 |  | 5.933 |  | 0.3136 |  |
| CPTSD |  | not about me |  | 155 |  | 15.55 |  | 11.461 |  | 0.9206 |  |
|  |  | not sure |  | 71 |  | 21.32 |  | 9.755 |  | 1.1577 |  |
|  |  | got to know |  | 33 |  | 22.88 |  | 8.623 |  | 1.5011 |  |
|  |  | work |  | 13 |  | 19.77 |  | 8.880 |  | 2.4629 |  |
|  |  | witnessing |  | 51 |  | 20.22 |  | 11.599 |  | 1.6242 |  |
|  |  | happened with me |  | 358 |  | 26.45 |  | 11.329 |  | 0.5988 |  |
|  | | | | | | | | | | | |

Table A3

Mean Differences for Anxiety, Depression, DSO, PTSD, and CPTSD according to the Reporting of Physical Violence

|  | | **LEC 5 physical violence** | | **N** | | **Mean** | | **SD** | | **SE** | |
| --- | --- | --- | --- | --- | --- | --- | --- | --- | --- | --- | --- |
| Anxiety |  | not about me |  | 310 |  | 2.065 |  | 1.677 |  | 0.0952 |  |
|  |  | not sure |  | 54 |  | 2.815 |  | 1.614 |  | 0.2197 |  |
|  |  | got to know |  | 72 |  | 2.653 |  | 1.809 |  | 0.2132 |  |
|  |  | work |  | 6 |  | 1.000 |  | 1.095 |  | 0.4472 |  |
|  |  | witnessing |  | 81 |  | 2.148 |  | 1.509 |  | 0.1677 |  |
|  |  | happened with me |  | 180 |  | 2.361 |  | 1.765 |  | 0.1315 |  |
| Depression |  | not about me |  | 310 |  | 2.174 |  | 1.778 |  | 0.1010 |  |
|  |  | not sure |  | 54 |  | 2.685 |  | 1.576 |  | 0.2145 |  |
|  |  | got to know |  | 72 |  | 2.917 |  | 1.701 |  | 0.2005 |  |
|  |  | work |  | 6 |  | 0.667 |  | 0.816 |  | 0.3333 |  |
|  |  | witnessing |  | 81 |  | 2.679 |  | 1.809 |  | 0.2009 |  |
|  |  | happened with me |  | 180 |  | 2.617 |  | 1.874 |  | 0.1397 |  |
| DSO |  | not about me |  | 310 |  | 10.603 |  | 7.581 |  | 0.4306 |  |
|  |  | not sure |  | 54 |  | 12.463 |  | 7.580 |  | 1.0315 |  |
|  |  | got to know |  | 72 |  | 12.722 |  | 6.826 |  | 0.8045 |  |
|  |  | work |  | 6 |  | 6.167 |  | 5.076 |  | 2.0723 |  |
|  |  | witnessing |  | 81 |  | 11.531 |  | 8.116 |  | 0.9018 |  |
|  |  | happened with me |  | 180 |  | 12.783 |  | 7.739 |  | 0.5768 |  |
| PTSD |  | not about me |  | 310 |  | 10.013 |  | 6.255 |  | 0.3553 |  |
|  |  | not sure |  | 54 |  | 11.889 |  | 6.436 |  | 0.8758 |  |
|  |  | got to know |  | 72 |  | 11.861 |  | 5.989 |  | 0.7058 |  |
|  |  | work |  | 6 |  | 8.833 |  | 2.317 |  | 0.9458 |  |
|  |  | witnessing |  | 81 |  | 11.185 |  | 6.539 |  | 0.7265 |  |
|  |  | happened with me |  | 180 |  | 11.911 |  | 5.850 |  | 0.4361 |  |
| CPTSD |  | not about me |  | 310 |  | 20.616 |  | 12.034 |  | 0.6835 |  |
|  |  | not sure |  | 54 |  | 24.352 |  | 12.139 |  | 1.6520 |  |
|  |  | got to know |  | 72 |  | 24.583 |  | 10.451 |  | 1.2317 |  |
|  |  | work |  | 6 |  | 15.000 |  | 6.132 |  | 2.5033 |  |
|  |  | witnessing |  | 81 |  | 22.716 |  | 12.067 |  | 1.3407 |  |
|  |  | happened with me |  | 180 |  | 24.694 |  | 11.750 |  | 0.8758 |  |
|  | | | | | | | | | | | |

Table A4

Mean Differences for Anxiety, Depression, Resilience, Stress, DSO, PTSD, and CPTSD according to the Reporting of Severe Human Suffering

|  | | **LEC 5 severe_suffering** | | **N** | | **Mean** | | **SD** | | **SE** | |
| --- | --- | --- | --- | --- | --- | --- | --- | --- | --- | --- | --- |
| Anxiety |  | not about me |  | 262 |  | 1.90 |  | 1.659 |  | 0.1025 |  |
|  |  | not sure |  | 99 |  | 1.87 |  | 1.489 |  | 0.1497 |  |
|  |  | got to know |  | 88 |  | 2.45 |  | 1.618 |  | 0.1725 |  |
|  |  | work |  | 20 |  | 2.25 |  | 1.860 |  | 0.4160 |  |
|  |  | witnessing |  | 100 |  | 2.41 |  | 1.658 |  | 0.1658 |  |
|  |  | happened with me |  | 134 |  | 3.01 |  | 1.743 |  | 0.1506 |  |
| Depression |  | not about me |  | 262 |  | 2.02 |  | 1.792 |  | 0.1107 |  |
|  |  | not sure |  | 99 |  | 2.19 |  | 1.582 |  | 0.1590 |  |
|  |  | got to know |  | 88 |  | 2.68 |  | 1.411 |  | 0.1504 |  |
|  |  | work |  | 20 |  | 2.30 |  | 1.809 |  | 0.4046 |  |
|  |  | witnessing |  | 100 |  | 2.51 |  | 1.806 |  | 0.1806 |  |
|  |  | happened with me |  | 134 |  | 3.31 |  | 1.893 |  | 0.1635 |  |
| Stress |  | not about me |  | 262 |  | 6.82 |  | 3.428 |  | 0.2118 |  |
|  |  | not sure |  | 99 |  | 7.41 |  | 2.766 |  | 0.2780 |  |
|  |  | got to know |  | 88 |  | 7.74 |  | 2.875 |  | 0.3065 |  |
|  |  | work |  | 20 |  | 7.80 |  | 2.802 |  | 0.6266 |  |
|  |  | witnessing |  | 100 |  | 7.96 |  | 2.974 |  | 0.2974 |  |
|  |  | happened with me |  | 134 |  | 8.59 |  | 2.902 |  | 0.2507 |  |
| Resilience |  | not about me |  | 262 |  | 2.99 |  | 0.862 |  | 0.0532 |  |
|  |  | not sure |  | 99 |  | 2.93 |  | 0.732 |  | 0.0735 |  |
|  |  | got to know |  | 88 |  | 2.70 |  | 0.791 |  | 0.0843 |  |
|  |  | work |  | 20 |  | 3.03 |  | 0.730 |  | 0.1632 |  |
|  |  | witnessing |  | 100 |  | 2.91 |  | 0.855 |  | 0.0855 |  |
|  |  | happened with me |  | 134 |  | 2.54 |  | 0.813 |  | 0.0703 |  |
| DSO |  | not about me |  | 262 |  | 9.69 |  | 7.734 |  | 0.4778 |  |
|  |  | not sure |  | 99 |  | 11.42 |  | 6.681 |  | 0.6715 |  |
|  |  | got to know |  | 88 |  | 12.84 |  | 7.045 |  | 0.7510 |  |
|  |  | work |  | 20 |  | 9.20 |  | 6.288 |  | 1.4060 |  |
|  |  | witnessing |  | 100 |  | 11.85 |  | 6.774 |  | 0.6774 |  |
|  |  | happened with me |  | 134 |  | 14.78 |  | 8.195 |  | 0.7079 |  |
| PTSD |  | not about me |  | 262 |  | 9.41 |  | 6.590 |  | 0.4071 |  |
|  |  | not sure |  | 99 |  | 10.76 |  | 4.891 |  | 0.4915 |  |
|  |  | got to know |  | 88 |  | 11.91 |  | 5.785 |  | 0.6167 |  |
|  |  | work |  | 20 |  | 10.95 |  | 4.751 |  | 1.0625 |  |
|  |  | witnessing |  | 100 |  | 11.32 |  | 6.145 |  | 0.6145 |  |
|  |  | happened with me |  | 134 |  | 13.24 |  | 6.010 |  | 0.5192 |  |
| CPTSD |  | not about me |  | 262 |  | 19.10 |  | 12.389 |  | 0.7654 |  |
|  |  | not sure |  | 99 |  | 22.18 |  | 9.366 |  | 0.9413 |  |
|  |  | got to know |  | 88 |  | 24.75 |  | 10.635 |  | 1.1337 |  |
|  |  | work |  | 20 |  | 20.15 |  | 9.354 |  | 2.0917 |  |
|  |  | witnessing |  | 100 |  | 23.17 |  | 11.102 |  | 1.1102 |  |
|  |  | happened with me |  | 134 |  | 28.01 |  | 12.135 |  | 1.0483 |  |
|  | | | | | | | | | | | |

Table A5

Mean Differences for Anxiety, Depression, Stress, DSO, PTSD, and CPTSD according to the Satisfaction by Current Living Conditions

|  | | | **Living Conditions** | | | | | | **N** | | | | | **Mean** | | | | | **SD** | | | | | **SE** | | | |  |
| --- | --- | --- | --- | --- | --- | --- | --- | --- | --- | --- | --- | --- | --- | --- | --- | --- | --- | --- | --- | --- | --- | --- | --- | --- | --- | --- | --- | --- |
| DSO |  | | very unsatisfactory | | |  | | 18 | |  | | 18.06 | | |  | | | 7.29 | | |  | | 1.718 | |  | |  |  |
|  |  | | unsatisfactory | | |  | | 30 | |  | | 18.30 | | |  | | | 7.47 | | |  | | 1.363 | |  | |  |  |
|  |  | | good enough | | |  | | 152 | |  | | 13.58 | | |  | | | 7.57 | | |  | | 0.614 | |  | |  |  |
|  |  | | satisfactory | | |  | | 392 | |  | | 10.64 | | |  | | | 7.22 | | |  | | 0.364 | |  | |  |  |
|  |  | | very satisfactory | | |  | | 89 | |  | | 9.33 | | |  | | | 7.44 | | |  | | 0.788 | |  | |  |  |
| PTSD |  | | very unsatisfactory | | |  | | 18 | |  | | 14.39 | | |  | | | 7.58 | | |  | | 1.786 | |  | |  |  |
|  |  | | unsatisfactory | | |  | | 30 | |  | | 15.00 | | |  | | | 7.09 | | |  | | 1.295 | |  | |  |  |
|  |  | | good enough | | |  | | 152 | |  | | 11.63 | | |  | | | 5.81 | | |  | | 0.472 | |  | |  |  |
|  |  | | satisfactory | | |  | | 392 | |  | | 10.53 | | |  | | | 5.89 | | |  | | 0.297 | |  | |  |  |
|  |  | | very satisfactory | | |  | | 89 | |  | | 10.04 | | |  | | | 6.95 | | |  | | 0.736 | |  | |  |  |
| CPTSD |  | | very unsatisfactory | | |  | | 18 | |  | | 32.44 | | |  | | | 11.38 | | |  | | 2.682 | |  | |  |  |
|  |  | | unsatisfactory | | |  | | 30 | |  | | 33.30 | | |  | | | 12.50 | | |  | | 2.282 | |  | |  |  |
|  |  | | good enough | | |  | | 152 | |  | | 25.20 | | |  | | | 11.45 | | |  | | 0.928 | |  | |  |  |
|  |  | | satisfactory | | |  | | 392 | |  | | 21.17 | | |  | | | 11.01 | | |  | | 0.556 | |  | |  |  |
|  |  | | very satisfactory | | |  | | 89 | |  | | 19.37 | | |  | | | 12.80 | | |  | | 1.357 | |  | |  |  |
| Anxiety | |  | | very unsatisfactory |  | | 18 | | | |  | | 3.28 | | |  | 1.565 | | |  | | 0.3688 | | |  | | | |
|  | |  | | unsatisfactory |  | | 30 | | | |  | | 3.17 | | |  | 1.913 | | |  | | 0.3493 | | |  | | | |
|  | |  | | good enough |  | | 152 | | | |  | | 2.52 | | |  | 1.830 | | |  | | 0.1485 | | |  | | | |
|  | |  | | satisfactory |  | | 392 | | | |  | | 2.07 | | |  | 1.614 | | |  | | 0.0815 | | |  | | | |
|  | |  | | very satisfactory |  | | 89 | | | |  | | 2.16 | | |  | 1.664 | | |  | | 0.1764 | | |  | | | |
| Depression | |  | | very unsatisfactory |  | | 18 | | | |  | | 4.61 | | |  | 1.335 | | |  | | 0.3146 | | |  | | | |
|  | |  | | unsatisfactory |  | | 30 | | | |  | | 3.63 | | |  | 1.829 | | |  | | 0.3339 | | |  | | | |
|  | |  | | good enough |  | | 152 | | | |  | | 2.68 | | |  | 1.854 | | |  | | 0.1504 | | |  | | | |
|  | |  | | satisfactory |  | | 392 | | | |  | | 2.27 | | |  | 1.703 | | |  | | 0.0860 | | |  | | | |
|  | |  | | very satisfactory |  | | 89 | | | |  | | 2.07 | | |  | 1.744 | | |  | | 0.1848 | | |  | | | |
| Stress | |  | | very unsatisfactory |  | | 18 | | | |  | | 11.11 | | |  | 3.008 | | |  | | 0.7089 | | |  | | | |
|  | |  | | unsatisfactory |  | | 30 | | | |  | | 10.50 | | |  | 2.623 | | |  | | 0.4789 | | |  | | | |
|  | |  | | good enough |  | | 152 | | | |  | | 7.87 | | |  | 3.109 | | |  | | 0.2522 | | |  | | | |
|  | |  | | satisfactory |  | | 392 | | | |  | | 7.16 | | |  | 2.983 | | |  | | 0.1507 | | |  | | | |
|  | |  | | very satisfactory |  | | 89 | | | |  | | 6.90 | | |  | 3.184 | | |  | | 0.3375 | | |  | | | |
|  | | | | | | | | | | | | | | | | | | | | | | | | | |  |  |  |

Table A6

Mean Differences for DSO, PTSD, and Stress according to the Current Living Location

|  | | | | **Current Location** | | **N** | | | | **Mean** | | | | | **SD** | | | | **SE** | | |  |  |
| --- | --- | --- | --- | --- | --- | --- | --- | --- | --- | --- | --- | --- | --- | --- | --- | --- | --- | --- | --- | --- | --- | --- | --- |
| DSO | |  | | The same place as before the war, never occupied |  | 379 | | |  | 10.72 | | |  | | 7.48 | |  | | 0.384 | |  |  |  |
|  | |  | | Moved to another country |  | 129 | | |  | 13.00 | | |  | | 8.04 | |  | | 0.708 | |  |  |  |
|  | |  | | Moved within Ukraine, officially registered IDP |  | 56 | | |  | 11.18 | | |  | | 6.48 | |  | | 0.865 | |  |  |  |
|  | |  | | Moved within Ukraine, did not officially registered as IDP |  | 52 | | |  | 13.65 | | |  | | 8.04 | |  | | 1.114 | |  |  |  |
|  | |  | | Moved to another country, did not officially register as refugee |  | 8 | | |  | 12.25 | | |  | | 9.27 | |  | | 3.277 | |  |  |  |
|  | |  | | Moved to another country, registered as refugee |  | 19 | | |  | 15.79 | | |  | | 7.69 | |  | | 1.763 | |  |  |  |
|  | |  | | The same place under occupation now |  | 7 | | |  | 9.14 | | |  | | 10.43 | |  | | 3.943 | |  |  |  |
|  | |  | | Other |  | 31 | | |  | 12.84 | | |  | | 6.82 | |  | | 1.225 | |  |  |  |
| ITQ_PTSD | |  | | The same place as before the war, never occupied |  | 379 | | |  | 11.08 | | |  | | 6.29 | |  | | 0.323 | |  |  |  |
|  | |  | | Moved to another country |  | 129 | | |  | 10.03 | | |  | | 5.58 | |  | | 0.491 | |  |  |  |
|  | |  | | Moved within Ukraine, officially registered IDP |  | 56 | | |  | 10.88 | | |  | | 6.18 | |  | | 0.826 | |  |  |  |
|  | |  | | Moved within Ukraine, did not officially registered as IDP |  | 52 | | |  | 11.33 | | |  | | 5.92 | |  | | 0.821 | |  |  |  |
|  | |  | | Moved to another country, did not officially register as refugee |  | 8 | | |  | 13.25 | | |  | | 7.03 | |  | | 2.484 | |  |  |  |
|  | |  | | Moved to another country, registered as refugee |  | 19 | | |  | 16.11 | | |  | | 7.20 | |  | | 1.652 | |  |  |  |
|  | |  | | The same place under occupation now |  | 7 | | |  | 13.57 | | |  | | 8.94 | |  | | 3.380 | |  |  |  |
|  | |  | | Other |  | 31 | | |  | 9.65 | | |  | | 5.44 | |  | | 0.978 | |  |  |  |
|  | | | | | | | | | | | | | | | | | | | | | |  |  |
| Stress |  | | The same place as before the war, never occupied | | | |  | 379 | | |  | 7.16 | |  | | 3.254 | |  | | 0.1671 | | |  |
|  |  | | Moved to another country | | | |  | 129 | | |  | 8.06 | |  | | 2.904 | |  | | 0.2557 | | |  |
|  |  | | Moved within Ukraine, officially registered IDP | | | |  | 56 | | |  | 7.32 | |  | | 2.943 | |  | | 0.3932 | | |  |
|  |  | | Moved within Ukraine, did not officially registered as IDP | | | |  | 52 | | |  | 8.37 | |  | | 3.081 | |  | | 0.4272 | | |  |
|  |  | | Moved to another country, did not officially registered as refugee | | | |  | 8 | | |  | 7.75 | |  | | 4.200 | |  | | 1.4850 | | |  |
|  |  | | Moved to another country, registered as refugee | | | |  | 19 | | |  | 8.89 | |  | | 2.447 | |  | | 0.5614 | | |  |
|  |  | | The same place under occupation now | | | |  | 7 | | |  | 7.57 | |  | | 3.994 | |  | | 1.5096 | | |  |
|  |  | | Other | | | |  | 31 | | |  | 8.00 | |  | | 2.875 | |  | | 0.5164 | | |  |
